# Better Visual Outcome by Intraocular Lens Ejection in Geriatric Patients with Ruptured Ocular Injuries

**DOI:** 10.1371/journal.pone.0170094

**Published:** 2017-01-20

**Authors:** Hiroki Kaneko, Tetsu Asami, Tadasu Sugita, Taichi Tsunekawa, Toshiyuki Matsuura, Kei Takayama, Kentaro Yamamoto, Shu Kachi, Yasuki Ito, Shinji Ueno, Norie Nonobe, Keiko Kataoka, Ayana Suzumura, Takeshi Iwase, Hiroko Terasaki

**Affiliations:** Department of Ophthalmology, Nagoya University Graduate School of Medicine, Nagoya, Japan; Massachusetts Eye & Ear Infirmary, Harvard Medical School, UNITED STATES

## Abstract

Ocular trauma is one of the leading causes of visual impairment worldwide. Because of the popularity of cataract surgeries, aged individuals with ocular trauma commonly have a surgical wound in their eyes. The purpose of this study was to evaluate the visual outcome of cases that were coincident with intraocular lens (IOL) ejection in the eyes with ruptured open-globe ocular injuries. Consecutive patients with open-globe ocular injuries were first reviewed. Patients’ characteristics, corrected distance visual acuities (CDVAs) over 3 years after the trauma, causes of injuries, traumatic wound patterns, and coexistence of retinal detachment were examined. The relationships between poor CDVA and the other factors, including the complications of crystalline lens and IOL ejection, were examined. A total of 105 eyes/patients [43 eyes with rupture, 33 with penetrating, 28 with intraocular foreign body (IOFB), and 1 with perforating injuries] were included. Rupture injuries were common in aged patients and were mostly caused by falls, whereas penetrating and IOFB injuries were common in young male patients. CDVAs of the eyes with rupture injuries were significantly worse than those of the eyes with penetrating or IOFB injuries. CDVA from more than 50% of the ruptured eyes resulted in no light perception or light perception to 20/500. CDVA of the ruptured eyes complicated by crystalline lens ejection was significantly worse than that of those complicated by IOL ejection. The wounds of the ruptured eyes complicated by IOL ejection were mainly located at the superior corneoscleral limbus, whereas those of the eyes complicated by crystalline lens ejection were located at the posterior sclera. There were significant correlations between poor CDVA and retinal detachment and crystalline lens ejection. These results proposed a new trend in the ocular injuries that commonly occur in aged patients; history of cataract surgery might affect the final visual outcome after open-globe ocular injuries.

## Introduction

Ocular trauma is one of the leading causes of visual impairment worldwide. Epidemiologic analysis has revealed that the cumulative lifetime prevalence of ocular trauma is approximately 20% [1]. In particular, open-globe injuries commonly cause severe visual impairment and blindness, at worst, and require immediate diagnosis and ophthalmologic intervention [2–6]. Feng et al. reported that a rupture, an open globe with a full-thickness wound more than 5 mm posterior to the corneoscleral limbus, a scleral wound of >10 mm, ciliary body damage, severe intraocular hemorrhage, a closed funnel retinal detachment (RD) or retinal prolapse, and choroidal damage are risk factors for no light perception (NLP) in eyes with a traumatized open-globe injury [7].

Although the types of injuries and severity of ocular trauma are totally different from those that occur in the battlefield, most ocular traumas predominantly occur in children and young males [8], which is probably due to the nature of children’s activity patterns, men’s occupations, etc. [9–11]. However, in their everyday clinical practice in urban areas, physicians noticed slight changes that have recently occurred. For example, the recent prevalence of ruptured open-globe injuries is distinct from what was reported a few decades ago. Ruptured open-globe injuries have become more prevalent in aged individuals regardless of sex. Because of the popularity of cataract surgeries, aged individuals with ocular trauma commonly have a surgical wound in their eyes, which may affect the pattern of ocular wounds and even the final outcome of treatment [12].

Therefore, we retrospectively studied cases of open-globe injuries and compared the prevalence and visual outcome in patients with ruptured eyes complicated by crystalline lens ejection and intraocular lens (IOL) ejection after undergoing cataract surgeries.

## Materials and Methods

### Patients and classifications

Data from patients with traumatic eye injuries who were hospitalized at the Department of Ophthalmology, Nagoya University Hospital, between August 1, 2003, and March 31, 2013 were retrospectively analyzed. Primary surgeries were performed either at Nagoya University Hospital (tertiary care hospital) or municipal hospitals (secondary medical care hospitals). According to our therapeutic protocol, multiple trauma surgeries were planned until the eyes showed better visual acuity. Secondary surgeries were performed within 7 days after the primary surgeries. Primary surgeries were performed mainly for closing all wounds, and secondary surgeries were performed for intraocular treatment. Intraocular surgeries were performed simultaneously only in cases wherein eyes developed mild or moderate damage. Age, sex, medical history, activity, cause, mechanism and complications of the injuries, including RD, therapeutic procedures, and best visual acuities under ophthalmic care over the 3 years after the injuries were recorded for each patient. Patients with factors that could affect the final visual outcome (i.e., severe glaucoma, history of corneal transplantation, inherited retinal diseases, and dementia) were excluded. The type of injury was classified based on the Birmingham Eye Trauma Terminology System [13]. The ocular trauma score (OTS) was calculated according to a previous report [14]. Ocular trauma was classified as closed (contusion and lamellar laceration) or open [rupture and penetrating, intraocular foreign body (IOFB), or perforating laceration] globe injury. In this study, only patients with open-globe injuries were evaluated. The eyes for which classification was difficult because of massive hemorrhage in the eyes or patients lacking information regarding the reason underlying the injuries were excluded. Because ocular traumas in cases of closed ocular injuries (e.g., commotio retinae) commonly receive treatment at a non-specialist hospital in our area, they were not referred to our hospital and we barely had an opportunity to follow them up [15]. All procedures conformed to the tenets of the World Medical Association’s Declaration of Helsinki. The Nagoya University Hospital Ethics Review Board approved this retrospective analysis of patient data.

### Wound pattern and lens status

The locations of the wounds in eyes with rupture injuries were recorded based on the surgical records and surgical videos. In addition, eyes with rupture injuries were divided into groups depending on the lens status: intact lens/IOL, traumatic cataract/lens dislocation, crystalline lens ejection, and IOL ejection. The zone of injuries in each group was classified according to a previous report [16].

### Best visual function after injuries

The corrected distance visual acuity (CDVA) over the 3 years after the injuries was defined as the best visual outcome in each eye. In this study, visual acuities were determined using a standard Landolt ring chart or Snellen chart. Both Landolt ring and Snellen charts are internationally recognized standards for visual acuity testing and are commonly used worldwide. The decimal scores obtained using Landolt ring charts are closely correlated with the fraction scores obtained using Snellen charts. Visual impairment was categorized based on the definition reported by the World Health Organization (**Table 1**) [17]. For statistical analysis, Landolt ring or Snellen ratings were converted to the logarithm of the minimum angle of resolution (LogMAR) scale. For non-Landolt ring/Snellen visual acuity, counting fingers, hand motion, light perception (LP), and NLP were assigned visual acuity (LogMAR) values of 20/4000 (2.301), 20/8000 (2.602), 20/16000 (2.903), and 20/32000 (3.204), respectively (**Table 2**) [18, 19]. Data were statistically analyzed using the Mann–Whitney *U* test (unpaired samples) or Pearson's chi-square test, and differences were considered to be statistically significant at *P* < 0.05. Multiple stepwise linear regression analysis was used to evaluate the correlation between the final logMAR visual acuity and independent variables such as age, sex, type of traumatic ocular injury, and the presence of RD, crystalline lens ejection, IOL ejection, traumatic cataract, or lens dislocation.

**Table 1:** Categorization of visual impairment.

Visual acuity	Category
20/60 or better	Normal or near-normal vision
20/400–20/70	Moderate or severe visual impairment
LP—20/500	Profound or near-total visual impairment
NLP	Total visual impairment

LP: Light perception, NLP: No light perception.

**Table 2:** Assignment of non-Landolt ring/Snellen acuity into logMAR values.

	Visual acuity (LogMAR)
Counting fingers	20/4000 (2.301)
Hand motion	20/8000 (2.602)
Light perception	20/16000 (2.903)
No light perception	20/32000 (3.204)

## Results

### Patient characteristics

**Fig 1:**
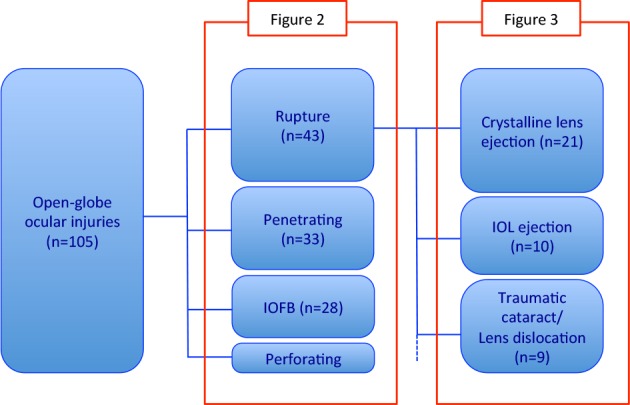
Schematic flow chart of this study.

Schematic flow chart of this study is shown in **Fig 1**and patient characteristics are shown in **Table 3**.

**Table 3:** Types of trauma and patients' ages.

	Total	Rupture	Penetrating	IOFB
Total	45.4±19.9	54.9±18.9	39.8±18.4	45.5±16.7
	(n = 105)	(n = 43)	(n = 33)	(n = 28)
Male	42.1±19.4	49.8±20.1	33.6±18.7	44.9±15.6
	(n = 87)	(n = 28)	(n = 31)	(n = 27)
Female	61.3±14.2	64.5±11.7	37.5±14.8	62
	(n = 18)	(n = 15)	(n = 2)	(n = 1)

IOFB: Intraocular foreign body.

We collected data from 121 cases which we could follow-up for 3 years after the trauma. Out of these 121 cases, 1 case with severe glaucoma, 3 cases with a history of corneal transplantation, 1 case with inherited retinal diseases, and 1 case with dementia were excluded. In total, 105 patients (105 eyes; 87 males and 18 females, all Japanese) underwent surgery for open-globe ocular injuries: 43 had ruptures, 33 had penetrating injuries, 28 had IOFB, and 1 had a perforating injury. Out of these 105 cases, primary surgeries for 61 cases were performed at Nagoya University Hospital (tertiary care hospital) and those for 34 cases were performed at municipal hospitals (secondary medical care hospitals), and all primary surgeries were performed within 24 h after trauma. There was no intraoperative complication in any patient. The mean patient age was 45.4 ± 19.9 years (males, 42.1 ± 19.4 years; females, 61.3 ± 14.2 years). Female patients, particularly those with ruptured eyes, were much older than the male patients. The mean patient age in the rupture group was 54.9 ± 18.9 years, which was much higher than that in the penetrating injury group. There were high percentages of male patients in the total (82.9%), rupture (65.1%), penetrating (93.9%), and IOFB (96.4%) groups. Similar to the results of many previous reports, we found that patients with ocular injuries were commonly young and male [1] [8] [10,11]. On the other hand, female patients were common among those with rupture injuries.

**Fig 2:**
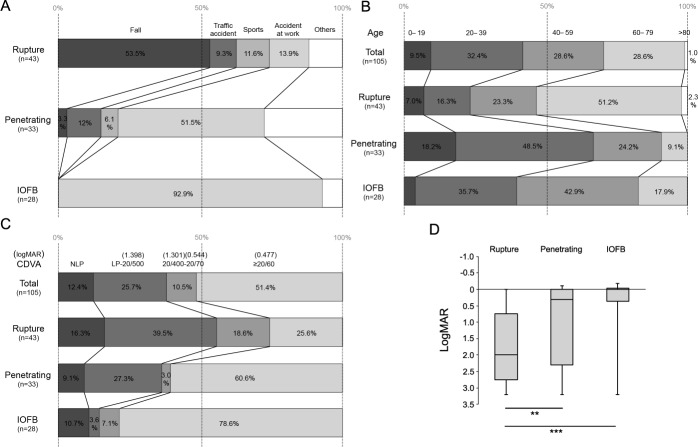
Main causes, age of the patients, and final visual outcome of ocular injuries. **(A)** Falls were the major cause (53.5%) of rupture injury. Accidents at work were major causes of penetrating injuries and IOFB, whereas falls were not or less relevant. **(B)** In the penetrating and IOFB groups, more patients were aged 0 to 19 years, 20 to 39 years, and 40 to 59 years, whereas in the ruptured ocular group, more patients were aged 60 years or older. **(C)** Among patients with penetrating and IOFB injuries, more than 60% of the eyes attained a visual acuity of 20/60 or more. On the other hand, more than 50% of the eyes with rupture injuries attained a final visual outcome of NLP or LP of 20/500. **(D)** There was a significant difference in the logarithm of the minimum angle of resolution (logMAR) values between ruptured eyes and eyes with penetrating or IOFB injuries. Error bars indicate whole range of distribution. **P = 0.0017, ***P < 0.001 IOFB, intraocular foreign body; LP, light perception; NLP, no light perception.

The main causes of the injuries are shown in **Fig 2A**. A fall was the most common cause of rupture injuries (23 of 43 eyes), whereas it was not a major factor in penetrating (only 1 of 33 eyes) and IOFB injuries. Instead, accidents at work were major causes of penetrating injuries (17 of 33 eyes) and IOFB (26 of 28 eyes).

The age distribution of the patients with open-globe ocular injuries is shown in **Fig 2B**. In total, the percentages of patients who were aged 20–39 years, 40 to 59 years, and 60 to 79 years were 32.4%, 28.6%, and 28.6%, respectively, and they were equally distributed. However, most patients with penetrating injuries and IOFB were aged 20–59 years. In contrast, more than 50% of the patients with ruptured eyes were aged 60 years or older.

The final visual outcomes within 3 years after the injuries (CDVA) are shown in **Fig 2C**. Visual acuity reached 20/60 or better in 78.6% of the eyes with IOFB and in more than 60% of the eyes with penetrating injuries. In contrast, only 25.6% of the eyes with rupture injuries showed 20/60 or better CDVA. In addition, more than 50% of the ruptured eyes had NLP or LP of 20/500. CDVA (logMAR) of the eyes with rupture injury (2.000, 0.747–2.753; median, Q1–Q3) was significantly worse than that of the eyes with penetrating (0.301, 0.000–2.301; median, Q1–Q3) or IOFB (0.000, −0.028–0.356; median, Q1–Q3; **Fig 2D**) injuries. These findings indicate that rupture injury leads to severe vision loss rather than eyes with penetrating or IOFB injuries.

### Wound pattern and visual outcome

**Fig 3:**
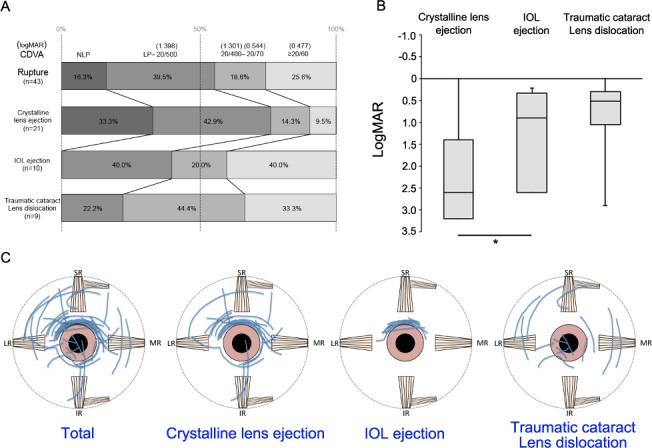
The difference in the final visual outcome depending on the lens damage. **(A)** More than 40% of the patients who had IOL ejection, traumatic cataract, or lens dislocation with ruptured ocular injuries showed a visual outcome of 20/400 or better. On the other hand, more than 70% of the patients with crystalline lens ejection had a visual outcome of 20/500 or worse. **(B)** A significantly better final visual outcome (logMAR) was attained in ruptured eyes complicated by IOL ejection than in those complicated by crystalline lens ejection. *P = 0.048. Error bars indicate whole range of distribution. **(C)** The wound patterns appeared to be different depending on whether the eyes had a crystalline lens (no history of cataract surgeries) or IOL (history of cataract surgeries). Among 43 ruptured eyes, the eyes complicated by crystalline lens ejection (*n* = 21) showed traumatic wounds at the corneoscleral limbus and deeper zone of the sclera. In contrast, the traumatic wounds of the eyes complicated by IOL ejection (*n* = 10) were mostly located only around the corneoscleral limbus. The wounds of the ruptured eyes complicated by traumatic cataract and lens dislocation (no lens/IOL ejection, *n* = 9) were not located around the corneoscleral limbus. Wounds in the left eyes were projected as a mirror image and sketched on the right eye. IOL, intraocular lens; logMAR, logarithm of the minimum angle of resolution.

We further analyzed the wound pattern of the eyes with rupture injuries by dividing the groups depending on the lens status (**Fig 3A**). Of the 43 eyes with rupture injuries, 21 were complicated by crystalline lens ejection, 10 by IOL ejection, and 9 by traumatic cataract, lens luxation, or lens dislocation. Only three eyes had an intact crystalline lens. The distribution of the ruptured eyes based on age is shown in **Table 4**.

**Table 4:** The distribution of the ruptured eyes in age.

	Number of eyes	Age				
		0–19	20–39	40–59	60–79	≥ 80
Rupture	43	3 (7.0%)	7 (16.3%)	10 (23.3%)	22 (51.2%)	1 (2.3%)
Crystalline lens ejection	21	2 (9.5%)	3 (14.3%)	7 (33.3%)	9 (42.9%)	0 (0%)
IOL ejection	10	0 (0%)	0 (0%)	0 (0%)	9 (90%)	1 (10%)
Traumatic cataract	9	1 (11.1%)	2 (22.2%)	2 (22.2%)	4 (44.4%)	0 (0%)
Lens dislocation						

IOL: Intraocular lens.

All the patients with IOL were 60-year old or older, whereas those with crystalline lens ejection were aged between 2 and 76 years. Among patients with crystalline lens ejection, there was no significant difference in the final visual outcome with an increase in age (data not shown).

OTSs of the ruptured eyes with crystalline lens ejection, IOL ejection, and traumatic cataract, lens luxation, or lens dislocation are shown in **Table 5**.

**Table 5:** Ocular trauma score in each group.

		Sum of raw points (mean ± SD)	Numbers of eyes (%)				
			OTS1	OTS2	OTS3	OTS4	OTS5
Rupture	Crystalline lens ejection	56.3 ± 16.0	7 (33.3%)	7 (33.3%)	6 (28.6%)	1 (4.8%)	0
	IOL ejection	69.5 ± 12.7	0	4 (40.0%)	5 (50.0%)	0	1 (10.0%)
	Traumatic cataract	68.1 ± 22.9	2 (22.2%)	2 (22.2%)	2 (22.2%)	2 (22.2%)	1 (11.1%)
	Lens dislocation						
Penetrating		64.9 ± 15.3	2 (6.1%)	16 (48.5%)	11 (33.3%)	2 (6.1%)	2 (6.1%)
IOFB		79.5 ± 20.8	2 (7.1%)	4 (14.3%)	7 (25.0%)	6 (21.4%)	8 (28.6%)

IOL: Intraocular lens, IOFB: Intraocular foreign body.

There were no IOL-implanted eyes with lens dislocation. Out of 21 eyes with crystalline lens ejection, one-third of the eyes resulted in NLP on CDVA, whileanother one-third had LP of 4/200 on CDVA. In contrast, none of the ruptured eyes complicated by IOL ejection resulted in NLP, 40.0% had LP to 20/500, and 40.0% had a CDVA of 20/60 or better. The CDVA (logMAR, **Fig 3B**) was significantly worse (*P* = 0.048) in the ruptured eyes complicated by crystalline lens ejection (2.602, 1.398–3.204; median, Q1–Q3) than that in ruptured eyes complicated by IOL ejection (0.898, 0.325–2.602; median, Q1–Q3). The wound patterns in the ruptured eyes complicated by crystalline lens ejection and IOL ejection appeared to be different (**Fig 3C**). The wounds in the ruptured eyes complicated by IOL ejection were predominantly located at the superior corneoscleral limbus, where most wounds for cataract surgeries are located. In contrast, the wounds in the ruptured eyes complicated by crystalline lens ejection were large and located not only at the superior corneoscleral limbus but also posterior to the plane of the incision of the four rectus muscles.

Zones of injuries and the number of surgeries in each group are listed and compared in **Table 6**.

**Table 6:** Zones of injuries and the number of surgeries.

		Zone			Number of surgeries	
		I	II	III	1	2 ≤
Rupture	Crystalline lens ejection	1	4	16	6	15
	IOL ejection	8	2	0	1	9
	Traumatic cataract	1	1	7	1	8
	Lens dislocation					
Penetrating		9	11	13	15	18
IOFB		12	8	8	20	8

IOL: Intraocular lens, IOFB: Intraocular foreign body.

### Retinal detachment as a complication of rupture injuries

We also investigated the involvement of RD complicated by ruptured ocular injuries (**S1 Fig**). Of the 43 eyes with ruptured ocular injuries, 36 (81.4%) had RD. In particular, 7 of the 10 (70.0%) ruptured eyes complicated by IOL ejection had RD, and 18 of the 21 (85.7%) ruptured eyes complicated by crystalline lens ejection had RD. There was no significant difference in the prevalence of RD between IOL-ejected eyes and crystalline lens-ejected eyes as a complication of ruptured ocular injuries (*P* = 0.30). However, results of multiple stepwise regression analysis testing the independence of the factors contributing to the final logMAR showed that the presence of crystalline lens ejection and RD were significantly correlated with poor CDVA, whereas the other factors did not show a significant correlation (**Table 7**).

**Table 7:** Results of multiple stepwise regression analysis for independence of factors contributing to the final logMAR visuals.

Variable			
Dependent	Independent	β	*P-*value
LogMAR	Retinal detachment	0.323	< 0.001
	Age	0.153	0.058
	Sex	-0.034	0.682
	Types of traumatic ocular injury	-0.142	0.128
	Crystalline lens ejection	0.391	< 0.001
	IOL ejection	0.125	0.139
	Traumatic cataract	-0.046	0.618
	Lens discolation		
	Zone	-0.118	0.22

IOL: Intraocular lens.

## Discussion

In this study, we focused not only on the general characteristics in the ruptured eyes but also on the difference of the visual outcome based on the difference in the lens complications.

With regard to the relationship between patients’ sex and the cause of the injuries, male patients comprised more than 50% of the total, ruptured, and penetrating injuries and IOFB. However, among these groups, there was less of a difference in the percentage of male and female patients in the rupture group. In patients with rupture injuries caused by a fall, the percentage of male versus female patients was 52.2% (n = 12) versus 47.8% (n = 11). Considering that traumatic injuries are more common in young males than females, the nearly 1:1 ratio of the patients pertaining to the sex in the group comprising patients with ocular injuries caused by a fall was very different from their ratio pertaining to the sex in the other groups. Andreoli et al. had similar findings in a large study of 848 patients wherein fall was the predominant cause of an open-globe injury in geriatric patients (103 of 166) and ruptured eyes comprised majority of geriatric injuries (143 of 166); among the younger population, males had injuries in a higher ratio than females, whereas among the geriatric population, this was not the case (nongeriatric males, 597; females 85 and geriatric males, 69; females, 97).[18]

IOFB is one of the main causes of severe vision loss. However, the rate of NLP was only 4% according to a previous report, which was much better than the final visual outcome after ruptured open-globe injuries [20]. In our study, the rate of NPL complicated by IOFB was 10.7%. However, we similarly concluded that IOFB injuries resulted in significantly better visual function than rupture injuries. Another previous retrospective study showed that 50% of eyes with open-globe injuries achieved a CDVA of 0.5 (20/40) or better [21]. In our study, 51.4% of the eyes with open-globe injuries achieved a visual acuity of 20/60 or better.

Among the different types of open-globe ocular injuries, rupture injury is one of the most severe injuries, as previously indicated.[22] To make matters worse, rupture injuries are very common in aged individuals. Our retrospective analysis revealed that among open-globe ocular injuries, a large number of aged patients had rupture injuries regardless of their sex. Our hospital is a tertiary care hospital located in one of the biggest cities in Japan, and we have never experienced traumatic cases caused by military attacks or gunshots. Instead, as shown in this study, we treated a certain percentage of aged female patients with ruptured eyes caused by a fall.

Our results showed that the ruptured eyes complicated by IOL ejection resulted in a better CDVA than those complicated by crystalline lens ejection. There was no significant difference between ruptured eyes with crystalline lens and IOL ejections with regard to the incident rates of RD (data not shown). However, the multiple stepwise regression analysis showed that crystalline lens ejection and RD were significantly correlated with poor CDVA. Deep scleral wounds located >5 mm posterior from the corneal limbus, presumably equal to the plane of insertion of the four rectus muscles, resulted in very poor final outcomes [6, 21, 23]. In addition, the wounds in the ruptured eyes complicated by crystalline lens ejection were large and located not only at the superior corneoscleral limbus but also at the posterior sclera (**Fig 3C**). Corroboration of these results indicated that the ruptured eyes with crystalline lens ejection were caused by extraordinarily high intraocular pressure, resulting in large traumatic wounds involving severe RD. In contrast, with regard to IOL-implanted eyes in which surgical wounds were made at the temporal-to-superior corneoscleral limbus, traumatic pressure was decreased by the opening of the pre-existing corneal scleral wound, thus avoiding the large traumatic wound from spreading to the posterior sclera.

One of the most important concerns in our study was whether the pre-existing corneoscleral wound truly avoided severe traumatic damage. It is difficult to fairly compare the wounds in the eyes with crystalline lens and those in the eyes with IOL when the initial injury/event is not 100% comparable. Some closed-globe injuries with a small traumatic impact might have resulted in unnecessary rupture injuries because of the re-opening of the pre-existing corneoscleral wound. While the wound may have predisposed to globe-ruptured trauma, it could have been protective in some way to allow for egress of intraocular material in a more controlled fashion. Because traumatic impact varies in each case, there are pros and cons pertaining to the existence of cataract wound in ocular trauma. The other concern is that we could not verify how many years ago the patients with IOL-ejection had cataract surgeries in their local area. These data are important for elucidating how the cataract wounds, e.g., length and place, affect the ruptured wounds. In addition, we did not have the medical records of the axial length of the injured eyes. Asian people more commonly have string myopia, which might affect the prevalence of traumatic retinal detachments. Another concern especially for ruptured open-globe injuries, which is our mainly targeted disease, is that we only had 43 eyes to be evaluated. Further analysis with larger number of samples could give us more detailed information for IOL-implanted eyes. In addition, in this study, we diagnosed only one eye with perforation. In eyes with massive hemorrhage, surgeons are sometimes unable to verify whether the scleral wounds are located not only in the anterior segment but also in the posterior segment of the eyes. Such eyes should be classified as having a perforation. However, in our study, we excluded some eyes in which the traumatic injury was difficult to classify.

Because of the improvement in medical technologies and the increased pursuit for a better quality of life, more aged individuals may opt for cataract surgery. Accordingly, we will have more opportunities, particularly in aged patients, to study traumatic ocular injuries with IOL complications. Our results may be helpful for understanding the changing trend of ocular injuries in future generations.

The visual outcome in patients with ruptured ocular injuries was worse than that in patients with other types of ocular injuries. However, the final visual outcome in patients with a ruptured eye complicated by IOL ejection was better than that in patients with a ruptured eye complicated by crystalline lens ejection. Presumably, this was because patients with rupture injuries who previously underwent cataract surgeries had injuries that were complicated by IOL ejection, with less frequent posterior scleral wounds and RDs.

## Supporting Information

S1 Fig The percentage of the existence of retinal detachment (RD) in patients with ruptured ocular injuries. There was no significant difference in the prevalence of RD between IOL-ejected eyes and crystalline lens-ejected eyes as a complication of ruptured ocular injuries (*P* = 0.30). IOL, intraocular lens; RD, retinal detachment
(EPS)

## References

[1] Wong TY, Klein BE, Klein R. The prevalence and 5-year incidence of ocular trauma: the Beaver Dam Eye Study. Ophthalmology. 2000;107(12):2196–202. 11097595 10.1016/s0161-6420(00)00390-0

[2] Chou C, Lou Y-T, Hanna E, Huang S-H, Lee S-S, Lai H-T, et al Diagnostic performance of isolated orbital CT scan for assessment of globe rupture in acute blunt facial trauma. Injury. 2016. 10.1016/j.injury.2016.01.014 26944178

[3] Shams-Vahdati S, Gholipour C, Jalilzadeh-Binazar M, Moharamzadeh P, Sorkhabi R, Jalilian R. Clinical findings provide criteria to evaluate priorities of ophthalmologic intervention in conscious multiple trauma patients. Injury. 2015;46(7):1238–40. 10.1016/j.injury.2014.10.056 25467708

[4] Sobaci G, Mutlu FM, Bayer A, KaragÜl S, Yildirim E. Deadly weapon–related open-globe injuries: outcome assessment by the Ocular Trauma Classification System. American journal of ophthalmology. 2000;129(1):47–53. 10653412 10.1016/s0002-9394(99)00254-8

[5] Savar A, Andreoli MT, Kloek CE, Andreoli CM. Enucleation for open globe injury. American journal of ophthalmology. 2009;147(4):595–600. e1. 10.1016/j.ajo.2008.10.017 19181305

[6] Rofail M, Lee GA, O’Rourke P. Prognostic indicators for open globe injury. Clinical & experimental ophthalmology. 2006;34(8):783–6. 17073902 10.1111/j.1442-9071.2006.01309.x

[7] Feng K, Hu YT, Ma Z. Prognostic indicators for no light perception after open-globe injury: eye injury vitrectomy study. American journal of ophthalmology. 2011;152(4):654–62. e2. 10.1016/j.ajo.2011.04.004 21726850

[8] Zhu L, Wu Z, Dong F, Feng J, Lou D, Du C, et al Two kinds of ocular trauma score for paediatric traumatic cataract in penetrating eye injuries. Injury. 2015;46(9):1828–33. 10.1016/j.injury.2015.04.024 25935359

[9] Rozga SR-P. Epidemiology of adult eye injuries in Split-Dalmatian county. Ophthalmology. 2004;45(3):304–9. 15185423

[10] May DR, Kuhn FP, Morris RE, Witherspoon CD, Danis R, Matthews G, et al The epidemiology of serious eye injuries from the United States Eye Injury Registry. Graefe's archive for clinical and experimental ophthalmology. 2000;238(2):153–7. 10766285 10.1007/pl00007884

[11] Katz J, Tielsch JM. Lifetime prevalence of ocular injuries from the Baltimore Eye Survey. Arch Ophthalmol-Chic. 1993;111(11):1564–8. 10.1001/archopht.1993.01090110130038 8240115

[12] Karimi A, Razaghi R, Navidbakhsh M, Sera T, Kudo S. Computing the stresses and deformations of the human eye components due to a high explosive detonation using fluid–structure interaction model. Injury. 2016. 10.1016/j.injury.2016.01.030 26861803

[13] Kuhn F, Morris R, Witherspoon CD, Heimann K, Jeffers JB, Treister G. A standardized classification of ocular trauma. Graefe's archive for clinical and experimental ophthalmology. 1996;234(6):399–403. 8738707 10.1007/BF00190717

[14] Kuhn F, Maisiak R, Mann L, Mester V, Morris R, Witherspoon CD. The ocular trauma score (OTS). Ophthalmology Clinics. 2002;15(2):163–5. 10.1016/s0896-1549(02)00007-x 12229231

[15] Williams DF, Mieler WF, Williams GA. Posterior segment manifestations of ocular trauma. Retina. 1990;10:S35–S44. 2191381 10.1097/00006982-199010001-00006

[16] Pieramici DJ, STERNBERG P, Aaberg TM, BRIDGES WZ, CAPONE A, Cardillo JA, et al A system for classifying mechanical injuries of the eye (globe). American journal of ophthalmology. 1997;123(6):820–31. 9535627 10.1016/s0002-9394(14)71132-8

[17] Maberley D, Hollands H, Chuo J, Tam G, Konkal J, Roesch M, et al The prevalence of low vision and blindness in Canada. Eye. 2006;20(3):341–6. 10.1038/sj.eye.6701879 15905873

[18] Andreoli MT, Andreoli CM. Geriatric traumatic open globe injuries. Ophthalmology. 2011;118(1):156–9. 10.1016/j.ophtha.2010.04.034 20709403

[19] Scott IU, Schein OD, West S, Bandeen-Roche K, Enger C, Folstein MF. Functional status and quality of life measurement among ophthalmic patients. Arch Ophthalmol-Chic. 1994;112(3):329–35. 10.1001/archopht.1994.01090150059023 8129657

[20] Szijártó Z, Gaál V, Kovács B, Kuhn F. Prognosis of penetrating eye injuries with posterior segment intraocular foreign body. Graefe's Archive for Clinical and Experimental Ophthalmology. 2008;246(1):161–5. 10.1007/s00417-007-0650-1 17674019

[21] Petroviè MG, Lumi X, Olup BD. Prognostic factors in open eye injury managed with vitrectomy: retrospective study. Croatian Medical Journal. 2004;45(3):299–303. 15185422

[22] Soni NG, Bauza AM, Son JH, Langer PD, Zarbin MA, Bhagat N. Open globe ocular trauma: functional outcome of eyes with no light perception at initial presentation. Retina. 2013;33(2):380–6. 10.1097/IAE.0b013e318263cefb 23026847

[23] Esmaeli B, Elner SG, Schork MA, Elner VM. Visual outcome and ocular survival after penetrating trauma: a clinicopathologic study. Ophthalmology. 1995;102(3):393–400. 7891976 10.1016/s0161-6420(95)31009-3

